# The Long-Term Impact of the COVID-19 Pandemic on the Prognosis of Patients With Acute Myocardial Infarction in Japan

**DOI:** 10.7759/cureus.77918

**Published:** 2025-01-24

**Authors:** Hiroki Sato, Keisuke Yonezu, Shotaro Saito, Ichitaro Abe, Katsunori Tawara, Hidefumi Akioka, Tetsuji Shinohara, Yasushi Teshima, Kunio Yufu, Ryuzo Abe, Tsuyoshi Shimomura, Naohiko Takahashi

**Affiliations:** 1 Department of Cardiology and Clinical Examination, Faculty of Medicine, Oita University, Yufu, JPN; 2 Department of Cardiology, Oita Red Cross Hospital, Oita, JPN; 3 Department of Emergency Medicine, Faculty of Medicine, Oita University, Yufu, JPN; 4 Department of Medical Informatics, Faculty of Medicine, Oita University, Yufu, JPN

**Keywords:** acute myocardial infarction, covid-19 pandemic, cox proportional hazards regression, kaplan-meier survival curves, major adverse cardiac event

## Abstract

Background: The prognosis of patients with acute myocardial infarction (AMI) in the initial stage of the coronavirus disease 2019 (COVID-19) outbreak has been reported globally. However, the reports on the prognosis of patients with AMI after the initial stage of the pandemic are limited worldwide.

Methods: This retrospective observational study utilized data from the electronic medical records system of the Oita University Hospital. This study encompassed patients who were hospitalized at our hospital for AMI treatment between April 2018 and June 2022. The study period was categorized into the following three periods: the pre-pandemic period (April 2018 to March 2020), the first phase of the pandemic (April 2020 to March 2021), and the second phase of the pandemic (April 2021 to June 2022). The primary outcome was the duration from the initial admission for AMI treatment to the onset of major adverse cardiac events (MACE). The secondary outcome was the duration from the initial admission for AMI treatment to death from any cause. These outcomes were compared among patients with AMI admitted during the three periods.

Results: The one-year MACE-free survival rates did not differ significantly among the three periods (p = 0.146), whereas the one-year overall survival rates were significantly different (p = 0.022). Univariate Cox regression analysis showed that patients with AMI had poorer overall survival in the second phase of the COVID-19 pandemic compared to the pre-pandemic period (hazard ratio {HR}: 4.92, 95% confidence interval {CI}: 1.27-19.09, p = 0.021). However, multiple regression analysis did not show significant differences in the overall survival of patients with AMI across the three periods (HR: 2.77, 95% CI: 0.59-12.96, p = 0.196).

Conclusion: The long-term impact of the COVID-19 pandemic on the prognosis of patients with AMI remains unclear. Further research is required to clarify the long-term impact by using larger cohorts that include pre-hospital data.

## Introduction

The coronavirus disease 2019 (COVID-19) pandemic has greatly impacted patients with acute myocardial infarction (AMI). In the initial stage of the COVID-19 pandemic, a reduction in AMI hospitalization has been reported worldwide [1-3]. Patients with AMI during the early pandemic phase tended to have worse severity and more complications [4]. The increased severity may have been caused by prolonged total ischemic time due to delays in transportation by emergency medical services during the pandemic [5]. Additionally, concerns regarding COVID-19 transmission may have caused hesitation in seeking emergency care, potentially leading to delays in AMI treatment [3]. Some studies have suggested that patients with ST-elevation myocardial infarction (STEMI) admitted during the pandemic had worse in-hospital mortality rates compared to the rates of those admitted during the pre-pandemic period [6-9]. However, other studies have not reported significant differences in in-hospital mortality among patients with AMI [10-12]. We also showed that the major adverse cardiac event (MACE)-free survival and overall survival rates of patients with AMI admitted during the first year of the COVID-19 pandemic (between April 2020 and March 2021) were not significantly different from those of patients admitted during the pre-pandemic period (between April 2018 and March 2020) [13]. The prognosis of patients with AMI in the initial stage of the COVID-19 pandemic has been reported globally [14]. Many studies have defined the early phase of the pandemic as the period of 2020. However, the reports on the prognosis of patients with AMI after the initial stage of the pandemic are limited worldwide.

In this study, we collected new data on patients with AMI admitted during the second year of the pandemic, aiming to compare their prognosis with that of patients with AMI admitted during the pre-pandemic period and the first year of the pandemic previously reported in this journal [13]. In Japan, the mass vaccination program for individuals of ≥ 65 years of age commenced in April 2021 [15]. By the end of July 2021, approximately 80% of this population had been administered a second dose of the vaccine [15]. Consequently, the second phase can be characterized as the phase in which the COVID-19 vaccination became widely administered. This study aimed to investigate the impact of the COVID-19 pandemic on patients with AMI in the second year when vaccines were widely administered.

## Materials and methods

Data collection and management

This retrospective observational study utilized data from the electronic medical records system of the Oita University Hospital. The study included patients hospitalized for AMI treatment at our hospital between April 2018 and June 2022. Patients were defined as having AMI based on the primary diagnosis, the admission-related diagnosis, or the diagnosis requiring the most resources, as recorded in the Japanese Diagnosis Procedure Combination database (DPC). The diagnosis of AMI was identified using the I21 code in the International Classification of Diseases-10 (ICD-10). Electronic medical records of patients with AMI during the study period were reviewed, and data on patient characteristics, laboratory results, medical history, coexisting diseases, and prognosis were collected.

The study period was categorized into the following three phases: the pre-pandemic period (April 2018 to March 2020), first phase of the pandemic (April 2020 to March 2021), and second phase of the pandemic (April 2021 to June 2022). On April 7, 2020, the Japanese government declared a state of emergency in response to the COVID-19 pandemic [16]. Therefore, in this study, April 2020 is defined as the beginning of the first phase of the pandemic. Other studies conducted in Japan have also defined April 2020 as the beginning of the pandemic [17,18].

Outcomes

The primary outcome was the duration from the initial admission for AMI treatment to the onset of MACE, defined as a composite of death from any cause, AMI, and stroke. The secondary outcome was the duration from the initial admission for AMI treatment to death from any cause, with follow-up conducted until June 2023. Patients who were alive 365 days after admission for AMI treatment were censored at 365 days.

Statistical analysis

Continuous variables are presented as mean with standard deviation or median with interquartile range and are compared by Student’s t-test or Wilcoxon rank sum test, as appropriate. Categorical variables are presented as frequency with percentage and are compared by Fisher’s exact test.

Kaplan-Meier plots were used to represent differences in survival among patients with AMI admitted during the following three periods: the pandemic's pre-pandemic, first, and second phases. A log-rank test was applied to evaluate patient prognoses across these periods. Univariate and multivariate Cox proportional hazards models were used to investigate the relationship between the prognosis of patients with AMI and the different phases of the pandemic. Variables with significant differences between the periods were included in the multivariate model as explanatory variables. Statistical significance was set at p < 0.05. All statistical analyses were performed using the R statistical software version 4.4.1 (Vienna, Austria: R Foundation for Statistical Computing).

## Results

Patient characteristics

**Figure 1:**
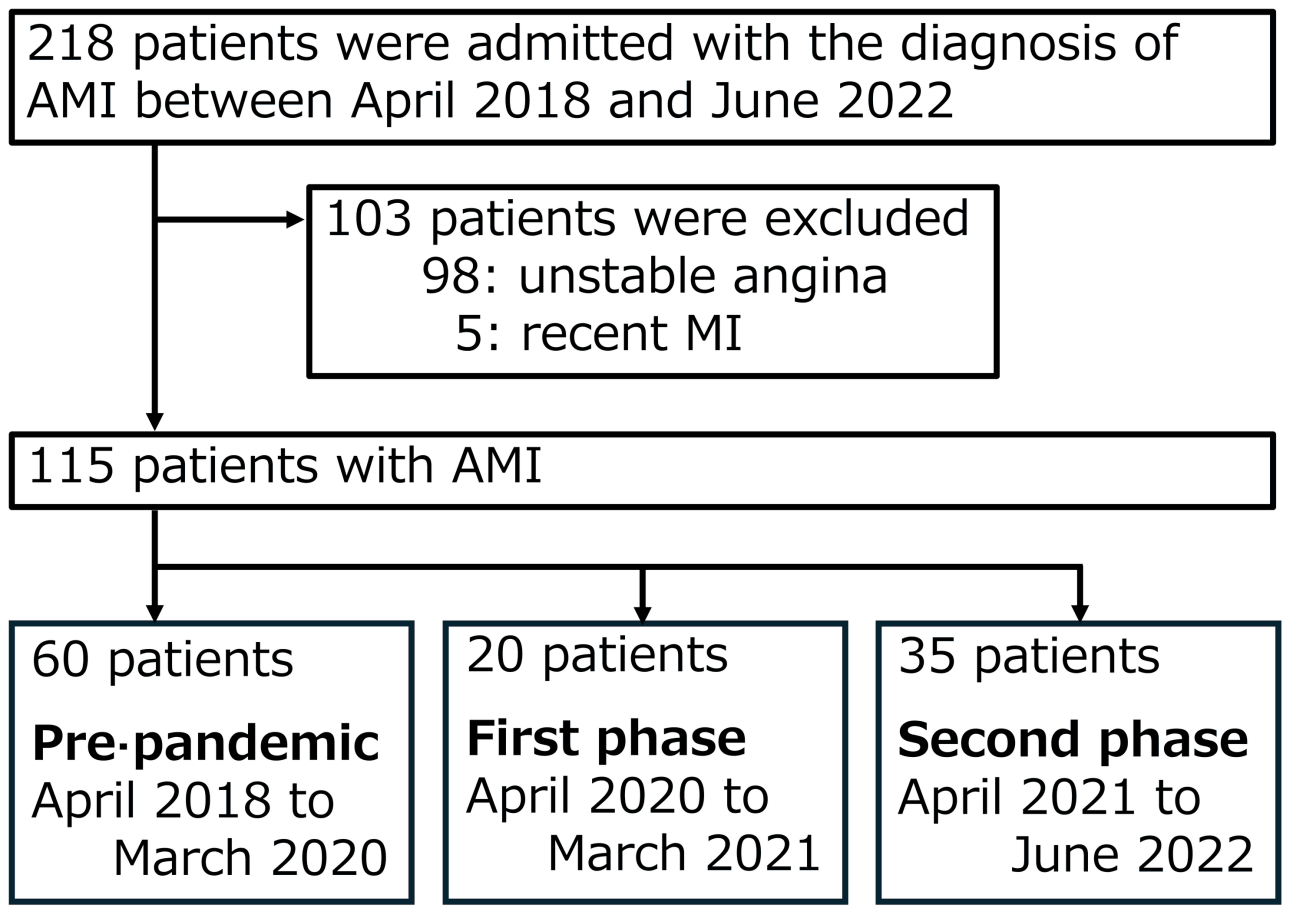
Flow diagram of the study population. Patients who were admitted for the treatment of AMI during the study period were enrolled. AMI: acute myocardial infarction; MI: myocardial infarction

In total, 115 patients admitted for the treatment of AMI during the study period were enrolled in this study, including 60, 20, and 35 patients admitted during the pandemic's pre-pandemic, first, and second phases, respectively (Figure 1).

The baseline patient characteristics are presented in Table 1 [13]. No missing data regarding the patient characteristics were reported. Significant differences were observed in high-density lipoprotein (HDL) cholesterol levels, the incidence of hypertension and dyslipidemia, and the prescription of mineralocorticoid receptor antagonists (MRAs) among patients across the three periods. The proportion of patients with STEMI was highest during the first phase of the pandemic. The levels of creatine kinase (CK) and CK-myocardial band (CK-MB) upon arrival at the hospital, peak CK, and N-terminal pro-B-type natriuretic peptide (NT-proBNP) were also highest among patients during the first phase of the pandemic. However, the differences were not statistically significant. None of the patients in the study population were infected with COVID-19 upon admission to the hospital.

**Table 1:** Baseline characteristics of patients with AMI. *P < 0.05 was considered statistically significant. Categorical data are represented as frequency (percentage). Continuous data are represented as mean±standard deviation or median (25 percentile, 75 percentile) as appropriate. AMI: acute myocardial infarction; STEMI: ST-elevation myocardial infarction; RCA: right coronary artery; LAD: left anterior descending artery; LCx: left circumflex artery; CK: creatine kinase; eGFR: estimated glomerular renal function; ACE-I: angiotensin-converting enzyme inhibitor; ARB: angiotensin receptor blocker; MRA: mineralocorticoid receptor antagonist; SGLT-2: sodium-glucose cotransporter-2; AMI: acute myocardial infarction; CK-MB: CK-myocardial band; LDL: low-density lipoprotein; HDL: high-density lipoprotein; NT-proBNP: N-terminal pro-B-type natriuretic peptide

Characteristic	Pre-pandemic (N = 60)	First phase (N = 20)	Second phase (N = 35)	p-Value
Male sex	47 (78.3)	14 (70.0)	27 (77.1)	0.762
Age (years)	71.7±11.6	77.9±11.5	73.1±11.2	0.114
STEMI	45 (75.0)	17 (85.0)	24 (68.6)	0.439
Culprit vessel				
RCA	18 (30.0)	7 (35.0)	13 (37.1)	0.534
LAD	36 (60.0)	9 (45.0)	18 (51.4)	
LCx	6 (10.0)	4 (20.0)	4 (11.4)	
Laboratory data				
CK, U/L	189.0 (116.0, 665.0)	241.5 (162.5, 436.0)	201.0 (108.0, 503.0)	0.857
CK-MB, ng/mL	15.9 (2.8, 63.2)	18.2 (6.3, 57.1)	5.45 (2.9, 59.3)	0.305
CK peak, U/L	1486.0 (722.0, 2845.0)	1822.0 (595.0, 3521.5)	1498 (390, 3392)	0.734
Troponin T, ng/mL	0.224 (0.040, 1.390)	0.142 (0.036, 0.528)	0.151 (0.03, 1.32)	0.614
LDL-cholesterol, mg/dL	121.7±40.2	100.4±40.6	111.3±31.6	0.081
HDL-cholesterol, mg/dL	48.3±13.0	51.8±15.6	60.5±23.0	0.005*
Creatinine, mg/dL	0.87 (0.72, 1.09)	0.89 (0.72, 1.31)	0.88 (0.76, 1.18)	0.796
eGFR, mL/min/1.73 m^2^	65.8±3.3	60.7±6.9	60.9±18.5	0.564
Hemoglobin, g/dL	13.6±2.0	13.0±2.0	13.5±2.1	0.430
NT-proBNP, pg/mL	361.2 (121.4, 1452.0)	2147.0 (201.0, 7697.0)	590.5 (107.0, 2931.0)	0.285
Risk factors for cardiovascular diseases				
Current smoker	17 (28.3)	4 (20.0)	10 (28.6)	0.663
Former smoker	24 (40.0)	6 (30.0)	11 (31.4)	
Hypertension	38 (63.3)	5 (25.0)	6 (17.1)	<0.001*
Dyslipidemia	35 (58.3)	10 (50.0)	10 (28.6)	0.017*
Diabetes mellitus	14 (23.3)	4 (20.0)	11 (31.4)	0.621
Chronic kidney disease	5 (8.3)	3 (15.0)	0 (0.0)	0.067
Length of hospital stay, days	20.1±3.4	19.5±1.8	22.3±14.2	0.945
Medical treatment at the discharge				
ACE-I/ARB	48 (80.0)	16 (80.0)	29 (82.9)	0.950
Beta-blocker	43 (71.7)	14 (70.0)	28 (80.0)	0.629
MRA	4 (6.7)	6 (30.0)	8 (22.9)	0.013*
SGLT-2 inhibitor	5 (8.3)	2 (10.0)	6 (17.1)	0.450
Statin	55 (91.7)	18 (90.0)	29 (82.9)	0.450

Prognosis of patients with AMI by the phase of the pandemic

**Figure 2:**
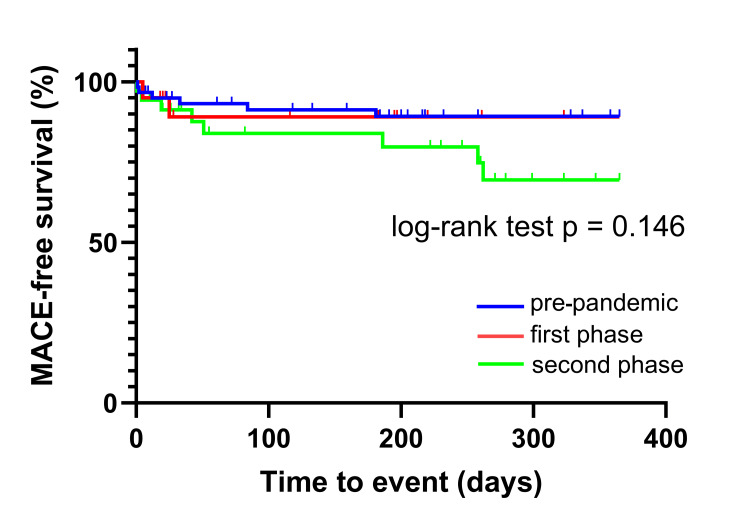
MACE-free survival of patients with AMI during the pre-pandemic period, as well as the first and second phases of the pandemic. MACE: major adverse cardiac event; AMI: acute myocardial infarction

**Figure 3:**
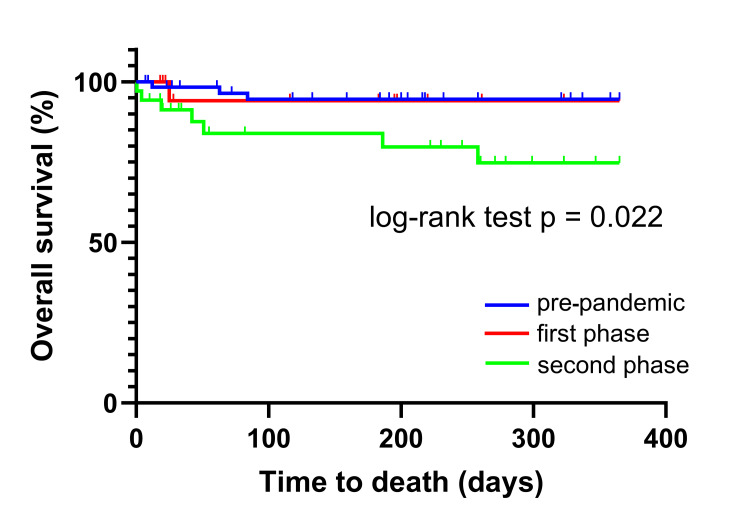
Overall survival of patients with AMI during the pre-pandemic period, as well as the first and second phases of the pandemic. AMI: acute myocardial infarction

The one-year MACE-free survival rate is shown in Figure 2. The one-year MACE-free survival rates among the three periods were not significantly different (p = 0.146). The one-year overall survival rates are shown in Figure 3, with significant differences observed between the pandemic phases (p = 0.022).

Significant differences were observed in HDL-cholesterol levels, prevalence of hypertension, and dyslipidemia, and prescription of MRA at discharge among the three periods. The HDL-cholesterol level was not included in the multiple regression model as an explanatory variable because it was strongly related to the prevalence of dyslipidemia. Similarly, MRA was not included in the model because MRA can be prescribed for hypertension. Therefore, the multiple regression model included the prevalence of hypertension and dyslipidemia as explanatory variables.

Univariate and multivariate Cox regression models for MACE-free survival showed no significant differences among the three periods (Table 2). The univariate Cox regression model showed significant differences in the overall survival of patients with AMI during the second phase compared with the pre-pandemic period (p = 0.021). However, the multiple regression analysis for overall survival did not show significant differences between the periods (p = 0.196).

**Table 2:** Cox proportional hazard regression analysis of patients with AMI. *P < 0.05 was considered statistically significant. Estimates of hazard ratio are represented as an estimated value with 95% CI. AMI: acute myocardial infarction; HR: hazard ratio; CI: confidence interval; AMI: acute myocardial infarction; MACE: major adverse cardiac event; COVID-19: coronavirus disease 2019

Outcomes	Univariate analysis		Multivariate analysis	
	HR (95% CI)	p-Value	HR (95% CI)	p-Value
MACE-free survival (COVID-19 pandemic)				
First phase	1.12 (0.23, 5.56)	0.890	0.89 (0.17, 4.61)	0.892
Second phase	2.64 (0.92, 7.63)	0.072	1.76 (0.53, 5.79)	0.353
Dyslipidemia	-	-	0.41 (0.12, 1.40)	0.157
Hypertension	-	-	0.69 (0.18, 2.60)	0.586
Overall survival (COVID-19 pandemic)				
First phase	1.11 (0.12, 10.71)	0.926	0.87 (0.09, 8.78)	0.907
Second phase	4.92 (1.27, 19.09)	0.021*	2.77 (0.59, 12.96)	0.196
Dyslipidemia	-	-	0.13 (0.02, 1.13)	0.064
Hypertension	-	-	0.72 (0.12, 4.29)	0.722

## Discussion

In this study, the prognosis of patients with AMI was compared between the pandemic's pre-pandemic, first, and second phases. The one-year MACE-free survival rates did not differ significantly across the three periods. The one-year overall survival of patients with AMI during the second phase of the pandemic was significantly higher than that of patients in the pre-pandemic period. However, the multiple-adjusted analysis showed no significant differences in overall survival among patients with AMI across the three periods.

A retrospective study of 98 patients with acute coronary syndrome admitted between March 2020 and March 2022 showed no significant difference in in-hospital mortality across the pandemic phases [11]. The in-hospital mortality of 598 patients with STEMI did not differ significantly across the pandemic's pre-pandemic period (March-August 2019), first year (March-August 2020), and second year (March-August 2021) [18]. These findings are consistent with the results of the present study.

In the present study, the multiple-adjusted analysis showed no significant differences in MACE-free survival or overall survival of patients with AMI across the three periods of the COVID-19 pandemic. These results suggest that the significant difference in overall survival rates among the three periods may be due to variations in patient characteristics. The multiple Cox regression model was adjusted for dyslipidemia and hypertension, both of which are known risk factors for AMI [19]. The poor prognosis of patients with AMI during the second phase could be partly explained by worsening lipid and blood pressure control due to the COVID-19 pandemic. However, the proportions of dyslipidemia and hypertension in patients with AMI during the second phase of the pandemic were relatively low, making it difficult to logically explain their relationship with the COVID-19 pandemic. The differences in the proportions of dyslipidemia and hypertension across the three periods may be attributed to random variations or unknown confounders. In the pre-pandemic phase, the prevalence of hypertension, a prognostic factor for AMI, was high. This may have contributed to the poorer prognosis of patients with AMI in the pre-pandemic phase; thus, the survival rates in the first and second phases, in which hypertension was relatively less prevalent, did not differ significantly. However, in the Cox regression analysis with hypertension as an adjustment variable, hypertension was not significant, suggesting that the higher prevalence of hypertension in the pre-pandemic phase may not have clearly impacted prognosis.

A retrospective study using data from the Japanese Registry of All Cardiac and Vascular Diseases Diagnosis Procedure Combination (JROAD-DPC), including 84,786 patients with AMI admitted between April 2019 and March 2021 in Japan, presented an increase in severity and overall 30-day mortality during the pandemic [18]. However, the adjusted 30-day mortality rate remained unchanged. In this study, although no significant differences were observed, the median levels of CK and CK-MB upon arrival at the hospital, peak CK, and NT-proBNP were highest among patients during the first phase of the pandemic (April 2020-March 2021). While MACE-free survival and overall survival did not differ between the pre-pandemic period and the first phase of the pandemic, these results may suggest a higher severity of AMI in patients during the first phase of the pandemic.

The strengths of this study are as follows: first, this study presents detailed information, including laboratory data and history, obtained by reviewing all the medical records of patients with AMI in the study population. Second, this study investigates the prognosis of patients with AMI after the first phase of the COVID-19 pandemic, which has not been adequately investigated. The results of this study are valuable in evaluating the long-term impact of the COVID-19 pandemic.

This study has several limitations. First, the sample size of this study may not be sufficient to detect differences in the survival of patients with AMI. Second, the generalizability of the study population is limited, as the study population included patients with AMI admitted to Oita University Hospital. The characteristics of patients with AMI in Oita Prefecture, such as severity, may differ from those of the general population. Third, factors such as delayed treatment or resource limitations due to the COVID-19 pandemic may have contributed to the results. However, data on these factors was not included in this study. Further, multiple regression analysis revealed no significant differences in survival among the three periods. These findings suggest that changes associated with the pandemic did not have a significant impact on the survival of patients with AMI.

## Conclusions

Our results show that the one-year MACE-free survival rates did not differ significantly across the three periods. The one-year overall survival of patients with AMI during the second phase of the pandemic was significantly higher than that of patients in the pre-pandemic period. However, the multiple-adjusted analysis showed no significant differences in overall survival among patients with AMI across the three periods. The findings of this study are valuable for considering treatment strategies for patients with AMI in case of future infectious disease outbreaks. The findings of this study are consistent with those of previous studies. The long-term impact of the COVID-19 pandemic on the prognosis of patients with AMI remains unclear. Further research is required to clarify the long-term impact by using larger cohorts, including pre-hospital data.
